# Post-marketing safety surveillance of sacituzumab govitecan: an observational, pharmacovigilance study leveraging FAERS database

**DOI:** 10.3389/fphar.2023.1283247

**Published:** 2023-11-10

**Authors:** Wensheng Liu, Qiong Du, Zihan Guo, Xuan Ye, Jiyong Liu

**Affiliations:** ^1^ Department of Pharmacy, Fudan University Shanghai Cancer Center, Shanghai, China; ^2^ Department of Oncology, Shanghai Medical College, Fudan University, Shanghai, China

**Keywords:** sacituzumab govitecan, antibody-drug conjugate, Trop-2, pharmacovigilance, adverse event, FAERS

## Abstract

**Background and objective:** Sacituzumab govitecan (SG), the first antibody-drug conjugate targeting human trophoblast cell-surface antigen 2 (Trop-2), has been approved by the Food and Drug Administration (FDA) for the treatment of advanced or metastatic breast cancer and urothelial cancer. However, there is currently a dearth of information regarding the safety profiles of SG in a large sample cohort. The objective of the present study is to investigate SG-related adverse events (AEs) in real-world settings leveraging the FDA Adverse Event Reporting System (FAERS) database to guide the safety management of clinical medication.

**Methods:** The FAERS database was retrospectively queried to extract reports associated with SG from April 2020 to March 2023. To identify and evaluate potential AEs in patients receiving SG, various disproportionality analyses such as reporting odds ratio (ROR), the proportional reporting ratio (PRR), the Bayesian confidence propagation neural network (BCPNN), and the multi-item gamma Poisson shrinker (MGPS) were employed.

**Results:** Overall, 2069 reports of SG as the “primary suspect” were identified. Noteworthy, SG was significantly associated with an increased risk of blood lymphatic system disorders (ROR, 7.18; 95% CI, 6.58–7.84) and hepatobiliary disorders (ROR, 2.68; 95% CI, 2.17–3.30) at the System Organ Class (SOC) level. Meanwhile, 61 significant disproportionality preferred terms (PTs) simultaneously complied with all four algorithms were adopted. Therein, anemia, thrombocytopenia, neutropenia, leukopenia, diarrhea, asthenia, alopecia, and electrolyte imbalance were consistent with the common AEs described in the clinical trials and specification of SG. Furthermore, unexpected significant AEs include colitis (ROR, 12.09; 95% CI, 9.1–16.08), heart rate increased (ROR, 5.11; 95% CI, 3.84–6.79), sepsis (ROR, 4.77; 95% CI, 3.59–6.34), cholestasis (ROR, 6.28; 95% CI, 3.48–11.36), blood bilirubin increased (ROR, 4.65; 95% CI, 2.42–8.94) and meningitis (ROR, 7.23; 95% CI, 2.71–19.29) were also be detected. The median time to onset of SG-related AEs was 14 [interquartile range (IQR), 7–52] days, with the majority occurring within the initial month of SG treatment.

**Conclusion:** Our study validates the commonly known AEs and also found some potentially emerging safety issues related to SG in real-world clinical practice, which could provide valuable vigilance evidence for clinicians and pharmacists to manage the safety issues of SG.

## 1 Introduction

Triple-negative breast cancer (TNBC) is a poor prognostic breast cancer subtype characterized by the lack of estrogen and progesterone receptors and the amplification of the human epidermal growth factor receptor 2 (HER2) gene, comprises approximately 15%–20% of invasive breast cancers (Garrido-Castro et al., 2019; Giaquinto et al., 2022). TNBC is associated with higher and earlier recurrence and mortality rates in the operable (I-III) stages and shorter overall survival (OS) in the inoperable (IV) stage (Leon-Ferre and Goetz, 2023). Although immunotherapy has shown promising benefits in first-line clinical treatment, systemic chemotherapy remains the mainstay of standard care for previously treated metastatic TNBC (mTNBC) (Twelves et al., 2016; Winer et al., 2021; Gradishar et al., 2023). However, in second-line or beyond mTNBC setting, single-agent chemotherapy has shown low response rates (10%–15%), short progression-free survival (PFS) (2–3 months), and significant toxicity (Twelves et al., 2016; Khosravi-Shahi et al., 2018; Park et al., 2019). To date, treatment options for mTNBC patients who have received two or more regimens remain limited. Hence, advances in therapeutic options for these breast cancer patients are urgently needed.

Trophoblast cell-surface antigen-2 (Trop-2), a transmembrane calcium signal transducer, is associated with poor outcome in multiple types of malignant epithelial tumors, including TNBC (Goldenberg et al., 2015; Kwapisz, 2022). Sacituzumab govitecan (SG) is an anti-Trop-2 antibody-drug coupling (ADC) consisting of a humanized Trop-2 antibody coupled to SN-38 (the active metabolite of irinotecan) via a proprietary, hydrolyzable linker (Goldenberg et al., 2015; Starodub et al., 2015; Kwapisz, 2022). In a phase 1/2, single-group, basket trial (IMMU-132-01, NCT01631552), clinical anti-tumor activity of SG monotherapy was first observed in the mTNBC patients (*n* = 108), with an objective response rate of 33.3%, a median PFS of 5.5 months, and median OS of 13.0 months (Bardia et al., 2021a). Subsequently, the clinical benefit of SG was further confirmed by the phase 3 ASCENT study (NCT02574455), which significantly prolonged median PFS (5.6 months vs. 1.7 months) and median OS (12.1 months vs. 6.7 months) in patients with heavily pretreated mTNBC, compared with single-agent chemotherapy of physician’s choice (Kwapisz, 2022; Bardia et al., 2021b; Carey et al., 2022; Kathpalia et al., 2023). Based on these impressive results, the Food and Drug Administration (FDA) approved SG (TRODELVY^®^) as one of the few targeted therapy options currently available for the treatment of mTNBC patients who have received at least two prior therapies (FDA grants regular approval to sacituzumab, 2021).

Despite that, with the knowledge of the SG payload is a cytotoxic ingredient, safety concerns should also be taken into consideration along with its efficacy (Carey et al., 2022). Nevertheless, with the widespread use of SG in clinical practice, limited available information on AEs associated with SG treatment, which mainly comes from clinical trials. According to the safety analyses reported in the previous clinical trial and the instructions of SG, the most common adverse events (AEs) of SG were neutropenia, diarrhea, alopecia, anemia, nausea, fatigue, constipation, and vomiting (Kathpalia et al., 2023; Bardia et al., 2017; Spring et al., 2021; Rugo et al., 2022a). However, the safety profiles of SG therapy in real-world, large sample cohort settings, in particular, time to onset of AEs associated with SG treatment have not been well elucidated to date.

Therefore, we conducted this pharmacovigilance study to evaluate the post-marketing safety profile of SG in real-world settings leveraging the FDA Adverse Event Reporting System (FAERS) database to provide vigilance reference for clinicians and pharmacists to manage the safety issues of SG.

## 2 Materials and methods

### 2.1 Study design and data source

This real-world, observational, retrospective pharmacovigilance study, performed from Quarter 2 (Q2) in 2020 to Q1 in 2023, was designed to explore SG related AEs leveraging the FAERS database. The FAERS database is a publicly accessible post-marketing safety surveillance database that including adverse event reports, product quality complaints, and medication error reports submitted by various occupational sources including health professionals, individual patients, pharmaceutical manufacturers, and lawyers (Fang et al., 2023). Despite FAERS is a US-centric database, it receives AE reports from around the global scope. Consequently, the extensive scale and worldwide reach of this open database render it highly appropriate for the evaluation of spontaneous reporting data. The FAERS database includes the following eight types of files: report sources (RPSR), demographic and administrative information (DEMO), drug information (DRUG), indications for use (INDI), start and end dates for reported drugs (THER), adverse events (REAC), patient outcomes (OUTC), and invalid reports (DELETED). All files are available on the FDA website (https://fis.fda.gov/extensions/FPD-QDE-FAERS/FPD-QDE-FAERS.html). Given that the FAERS databases are accessible to the public and patient records are anonymized and de-identified, neither informed consent nor ethical approval was involved.

### 2.2 Data extraction and mining

**FIGURE 1:**
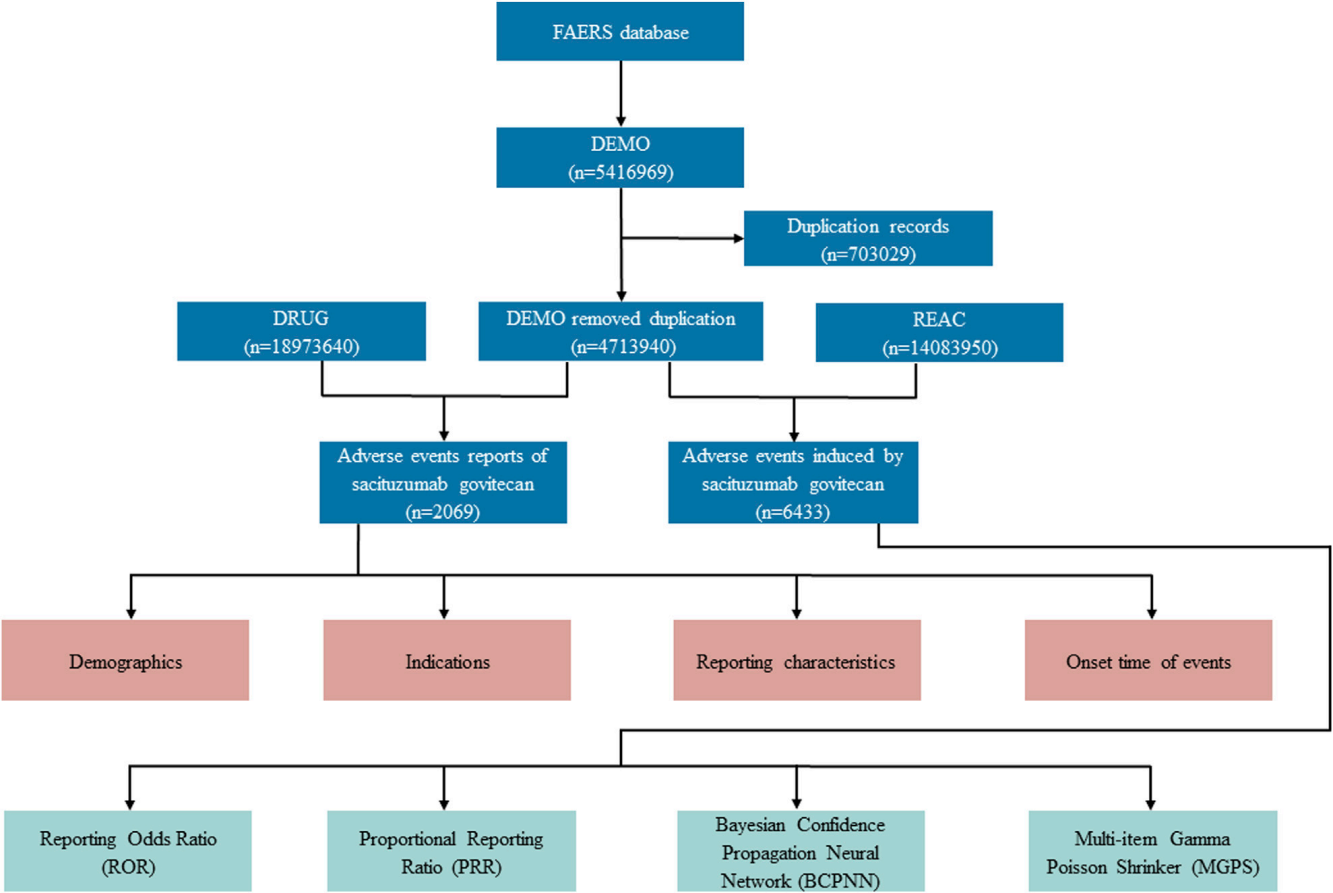
Flow diagram for the selection of AEs associated with sacituzumab govitecan from FAERS database.

The data extraction and mining of the present study is illustrated in Figure 1. Generic names and brand names (sacituzumab govitecan, Trodelvy^®^) were applied to identify SG-related reports due to two variables, PROD_AI and DRUGNAME. Besides, in the light of FDA’s recommendations, we eliminated duplicate reports filed by different people and institutions by choosing the latest FDA_DT when the PRIMARYIDs were the same, and the higher PRIMARYID where the FDA_DT and the CASEID were the same. Generally, drugs reported within FAERS were categorized into four patterns: primary suspect (PS), secondary suspect (SS), concomitant (C), and interacting (I). In our investigations, exposure assessment was only considered when SG was documented as “primary suspect.” The AE reports in FAERS database are coded according to Preferred Terms (PTs) in the Medical Dictionary for Regulatory Activities (MedDRA). The hierarchical structure of MedDRA allows for PTs to be categorized into the relevant System Organ Class (SOC), which is the highest level of MedDRA. Clinical characteristics, including demographics (age, gender), reporting characteristics (reporting year, region, and occupation of reporters), and indications of reports were collected. Meanwhile, serious outcomes were defined as death, life-threatening, hospitalization (initial or prolonged), disability, congenital anomaly, or other important medical event. Additionally, the time to onset of specific AEs induced by SG was also assessed, calculated as the interval between the time of SG dosage initial (START_DT) and the time of AE onset (EVENT_DT) (Shu et al., 2023a). Reports with dates missing or incorrect (drug usage time later than the time of event occurrence) were excluded.

### 2.3 Statistical analysis

Descriptive analysis was used to characterize all AEs reports in relation to SG treatment. In our investigations, both frequentist methods [reporting odds ratio (ROR) (van Puijenbroek et al., 2002) and proportional reporting ratio (PRR) (Evans et al., 2001)] and Bayesian methods [information component (IC) (Bate et al., 1998) and empirical Bayes geometric mean (EBGM) (Szarfman et al., 2002)] of disproportionality analysis were applied to identify the potential AE signals associated with SG, as a way to confirm our findings and reduce false-positive safety signals. A two-by-two contingency table is the framework for analyses (Supplementary Table S1). Besides, detailed equations and criteria for the four algorithms are presented in Table 1. In the present study, drug-related AE signals were identified based on the inclusion of signals with a minimum of three AE records associated with target drugs, and only AE signals that simultaneously met all four algorithm standards aforementioned were deemed as significant positive indicators (Shu et al., 2023b). All data processing and statistical analyses were performed using SAS 9.4 (SAS Institute Inc., Cary, NC, United States), Microsoft EXCEL Professional Plus 2013, and the GraphPad Prism 8.0 (GraphPad Software, CA, United States).

**TABLE 1:** Four major algorithms used for signal detection.

Algorithms	Calculation formulas	Criteria
ROR	$\text{ROR}=\frac{\left(a/c\right)}{\left(b/d\right)}=\frac{ad}{bc}$	95%CI > 1, a≥3
	$95\%\text{CI}=e^{\ln\left(\text{ROR}\right)\pm 1.96\sqrt{\left(\frac{1}{a}+\frac{1}{b}+\frac{1}{c}+\frac{1}{d}\right)}}$	
PRR	$\text{PRR}=\frac{a/\left(a+b\right)}{c/\left(c+d\right)}$	PRR≥2, χ^2^ ≥ 4, a≥3
	$\chi 2=\frac{{\left(\text{ad}-\text{bc}\right)}^{2}\left(a+b+c+d\right)}{\left(a+b\right)\left(a+c\right)\left(c+d\right)\left(b+d\right)}$	
BCPNN	IC = $log_{2}\frac{a\left(a+b+c+d\right)}{\left(a+b\right)\left(a+c\right)}$	IC025 > 0
	E (IC)= $log_{2}\frac{\left(a+\gamma 11\right)\left(a+b+c+d+\alpha \right)\left(a+b+c+d+\beta \right)}{\left(a+b+c+d+\gamma \right)\left(a+b+\alpha 1\right)\left(a+c+\beta 1\right)}$	
	V(IC) = $\frac{1}{{\left(\ln2\right)}^{2}}\left\{\left[\frac{\left(a+b+c+d\right)-a+\gamma -\gamma 11}{\left(a+\gamma 11\right)\left(1+a+b+c+d+\gamma \right)}\right]+\left[\frac{\left(a+b+c+d\right)-\left(a+b\right)+\alpha -\alpha 1}{\left(a+b+\alpha 1\right)\left(1+a+b+c+d+\alpha \right)}\right]+\left[\frac{\left(a+b+c+d\right)-\left(a+c\right)+\beta -\beta 1}{\left(a+c+\beta 1\right)\left(1+a+b+c+d+\beta \right)}\right]\right\}$	
	$\gamma =\gamma 11\frac{\left(a+b+c+d+\alpha \right)\left(a+b+c+d+\beta \right)}{\left(a+b+\alpha 1\right)\left(a+c+\beta 1\right)}$	
	IC025*=E(IC)-2* $\sqrt{V\left(IC\right)}$	
MGPS	$\text{EBGM}=\frac{a\left(a+b+c+d\right)}{\left(a+c\right)\left(a+b\right)}$	EBGM05 > 2
	$95\%\text{CI}=e^{\ln\left(\text{EBGM}\right)\pm 1.96\sqrt{\left(\frac{1}{a}+\frac{1}{b}+\frac{1}{c}+\frac{1}{d}\right)}}$	

Abbreviation: ROR, reporting odds ratio; PRR, proportional reporting ratio; BCPNN, bayesian confidence propagation neural network; MGPS, multi-item gamma Poisson shrinker; EBGM, empirical Bayesian geometric mean; CI, confidence interval; χ^2^, chi-squared; IC, information component; IC025, the lower limit of the 95% one-sided CI, of the IC; EBGM05, the lower 95% one-sided CI, of EBGM. Equation: a, number of reports containing both the target drug and the target adverse events; b, number of reports containing the target adverse drug reaction with other medications (except the target drug); c, number of reports containing the target drug with other adverse events (except the target adverse events); d, number of reports containing other medications and other adverse events.

## 3 Results

### 3.1 Descriptive analysis

During the surveillance period, from April 2020 to March 2023, a total of 4,713,940 reports were documented in the FAERS database, and 2069 (0.04%) reports were associated with SG medication [patient median (interquartile range, IQR) age, 56 (46–66) years]. The specific demographic and clinical details are provided in Table 2. Gender data were available for 1,950 patients, and the proportion of women was 85.40%. Therein, middle-aged patients (18–65 years) tended to have a higher risk of SG related AEs (*n* = 732, 35.38%). Furthermore, a significant proportion of patients (*n* = 1,845, 89.17%) experienced serious outcomes, including hospitalizations (616 cases), deaths (544 cases), and life-threatening situations (108 cases) with available follow-up data. From the perspective of reporting sources, 1,823 healthcare professionals-including doctors (52.15%) and pharmacists (35.69%)-submitted 88.11% of the AE reports, as compared to 245 consumers who reported 11.84% of the AEs, and 1unknown person who reported 0.05% of the AEs. According to the data presented in Table 2, the United States of America reported the most number of AE cases, with a total of 744, accounting for 35.96% of the whole. This was followed by France (*n* = 387, 18.70%), and Canada (*n* = 245, 11.84%). Besides, the number of reported AEs-related to SG showed a gradual increase from 2020 to 2023. However, it is worth noting that except for 60.75% reported in 2022, the most reported year was the first quarter of 2023 (18.76%).

**TABLE 2:** Summary of basic demographic and clinical information of reports associated with sacituzumab govitecan based on the FAERS database (From 1 April 2020 to 31 March 2023).

Characteristics	Case number, n	Case proportion, %
Number of events	2,069	
Gender		
Female	1,767	85.40
Male	183	8.84
Unknown	119	5.75
Age		
<18	0	0.00
18≤ and <45	227	10.97
45≤ and <65	505	24.41
≥65	276	13.34
Unknown	1,061	51.28
Indications (Top Five)		
Triple negative breast cancer	1,046	50.56
Product used for unknown indication	388	18.75
Breast cancer metastatic	145	7.01
Breast cancer	118	5.70
Transitional cell carcinoma	74	3.58
AE Severity		
Serious	1,845	89.17
Non-serious	224	10.83
Serious Outcome		
Death	544	26.29
Life-Threatening	108	5.22
Hospitalization - Initial or Prolonged	616	29.77
Disability	27	1.30
Required Intervention to Prevent Permanent Impairment	2	0.10
Other serious medical events	1,506	72.29
Reporting Year		
2020	77	3.72
2021	359	17.35
2022	1,257	60.75
2023Q1	376	18.17
Reported Countries (Top Five)		
United States of America	744	35.96
France	387	18.70
Canada	245	11.84
Germany	82	3.96
Italy	82	3.96
Reported Person		
Physician	1,079	52.15
Pharmacist	744	35.96
Consumer	245	11.84
Unknown	1	0.05
Time to onset of SG related AEs		
0-30d	533	25.76
31-90d	145	7.00
91-180d	83	4.01
181-360d	42	2.03
>360d	11	0.53

AE, adverse event; 2023Q1, the first quarter of 2023.

### 3.2 AE profiling of sacituzumab govitecan in disproportionality analysis

**FIGURE 2:**
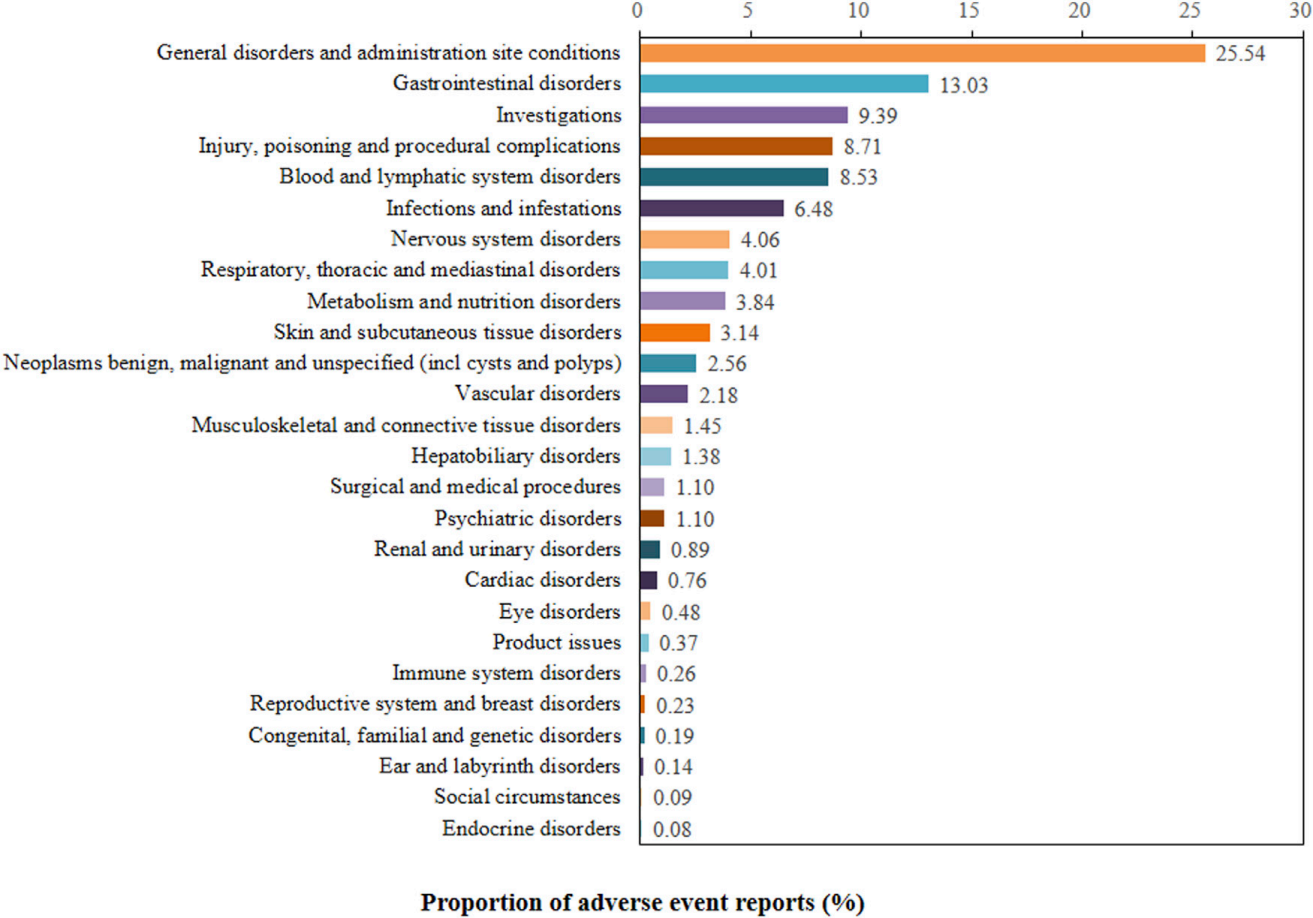
Proportion of positive signal for sacituzumab govitecan related AEs at the System Organ Class (SOC) level.

The proportion of positive signals for SG related AEs at the SOC level were shown in Figure 2. Meanwhile, specific signal strength of SG at the SOC level were described in Table 3. Statistically, we identified 26 organ systems that were involved in SG-induced AEs. In particular, the significant SOCs that met four criteria were investigations (SOC: 10022891, *n* = 604), blood and lymphatic system disorders (SOC: 10005329, *n* = 549), metabolism and nutrition disorders (SOC: 10027433, *n* = 247), hepatobiliary disorders (SOC: 10019805, *n* = 89), and congenital, familial and genetic disorders (SOC: 10010331, *n* = 12). Since FAERS is a collection of all medical and health-related PTs, it will also contain some non-drug-related AE signals that may be caused by disease progression or other causes. Therefore, in the present study, neoplasms benign, malignant and unspecified (incl cysts and polyps) (SOC: 10029104), injury, poisoning and procedural complications (SOC: 10022117), product issues (SOC: 10077536), surgical and medical procedures (SOC: 10042613) were not included for further analyses of SG-related AEs (Supplementary Table S2).

**TABLE 3:** The signal strength of reports associated with sacituzumab govitecan at the system organ class (SOC) level in FAERS database.

SOC	Case number (n)	ROR (95% CI)	PRR (χ2)	IC (IC025)	EBGM (EBGM05)
General disorders and administration site conditions	1643	2.04 (1.93–2.16)	1.78 (650.36)	0.83 (0.75)	1.78 (1.68)
Gastrointestinal disorders	838	2.24 (2.09–2.41)	2.08 (502.30)	1.06 (0.95)	2.08 (1.94)
Investigations	604	**2.67 (2.46–2.91)**	**2.52 (573.22)**	**1.33 (1.20)**	**2.52 (2.31)**
Injury, poisoning and procedural complications	560	1.01 (0.93–1.10)	1.01 (0.06)	0.01 (−0.11)	1.01 (0.93)
Blood and lymphatic system disorders	549	**7.18 (6.58–7.84)**	**6.65 (2662.35)**	**2.73 (2.59)**	**6.63 (6.08)**
Infections and infestations	417	1.61 (1.46–1.78)	1.57 (90.41)	0.65 (0.50)	1.57 (1.42)
Nervous system disorders	261	0.77 (0.68–0.87)	0.78 (17.91)	−0.37 (−0.55)	0.78 (0.69)
Respiratory, thoracic and mediastinal disorders	258	1.17 (1.04–1.33)	1.17 (6.31)	0.22 (0.04)	1.17 (1.03)
Metabolism and nutrition disorders	247	**3.03 (2.67–3.44)**	**2.95 (321.95)**	**1.56 (1.36)**	**2.95 (2.59)**
Skin and subcutaneous tissue disorders	202	0.85 (0.74–0.98)	0.85 (5.37)	−0.23 (−0.44)	0.85 (0.74)
Neoplasms benign, malignant and unspecified (incl cysts and polyps)	165	1.72 (1.48–2.01)	1.70 (48.74)	0.77 (0.54)	1.70 (1.46)
Vascular disorders	140	1.68 (1.42–1.99)	1.67 (37.85)	0.74 (0.48)	1.67 (1.41)
Musculoskeletal and connective tissue disorders	93	0.48 (0.39–0.59)	0.49 (50.79)	−1.03 (−1.32)	0.49 (0.40)
Hepatobiliary disorders	89	**2.68 (2.17–3.30)**	**2.65 (91.96)**	**1.41 (1.07)**	**2.65 (2.15)**
Surgical and medical procedures	71	1.42 (1.13–1.80)	1.42 (8.83)	0.50 (0.15)	1.42 (1.12)
Psychiatric disorders	71	0.47 (0.37–0.60)	0.48 (41.34)	−1.06 (−1.40)	0.48 (0.38)
Renal and urinary disorders	57	0.82 (0.63–1.06)	0.82 (2.25)	−0.28 (−0.66)	0.82 (0.63)
Cardiac disorders	49	0.65 (0.49–0.87)	0.66 (8.93)	−0.61 (−1.01)	0.66 (0.50)
Eye disorders	31	0.48 (0.34–0.69)	0.49 (17.04)	−1.04 (−1.53)	0.49 (0.34)
Product issues	24	0.80 (0.53–1.19)	0.80 (1.21)	−0.32 (−0.89)	0.80 (0.54)
Immune system disorders	17	0.37 (0.23–0.59)	0.37 (18.45)	−1.44 (−2.07)	0.37 (0.23)
Reproductive system and breast disorders	15	3.21 (1.94–5.34)	3.21 (22.79)	1.68 (0.77)	3.21 (1.93)
Congenital, familial and genetic disorders	12	**50.02 (28.21–88.68)**	**49.92 (562.52)**	**5.61 (2.57)**	**48.83 (27.54)**
Ear and labyrinth disorders	9	0.67 (0.35–1.28)	0.67 (1.52)	−0.59 (−1.45)	0.67 (0.35)
Social circumstances	6	0.46 (0.21–1.02)	0.46 (3.83)	−1.12 (−2.10)	0.46 (0.21)
Endocrine disorders	5	0.79 (0.33–1.90)	0.79 (0.28)	−0.34 (−1.47)	0.79 (0.33)

Note: Values in bold indicates significant signals in four algorithms. PRR, proportional reporting ratio; ROR, reported odds ratio; IC, information component; EBGM, the empirical Bayes geometric mean; IC025 and EBGM05, lower limit of the 95% two-sided confidence interval for IC and EBGM, respectively. Signals are detected when all the following criteria are met: PRR ≥ 2 and χ2 > 4, lower limit of 95% CI of ROR > 1, IC025 > 0, EBGM025 > 2.

Furthermore, a total of 61 significant disproportionality PTs that simultaneously comply with the four algorithms is shown in Table 4. In the present study, PTs of neutropenia (PT: 10029354), febrile neutropenia (PT: 10016288), anaemia (PT: 10002034), thrombocytopenia (PT: 10043554), periorbital oedema (PT: 10034545), diarrhoea (PT: 10012735), leukopenia (PT: 10024384), asthenia (PT: 10003549), mucosal inflammation (PT: 10028116), hepatic lesion (PT: 10061998), cholinergic syndrome (PT: 10008674), pleural effusion (PT: 10035598), pneumonitis (PT: 10035742), alopecia (PT: 10001760), hypokalaemia (PT: 10021015), lymphoedema (PT: 10025282) were detected, which were consistent with findings from clinical trials and the label for SG. Noteworthy, some unexpected AEs uncovered in the label of SG were also founded, including colitis (PT: 10009887), heart rate increased (PT: 10019303), cholecystitis acute (PT: 10008614), hepatic cytolysis (PT: 10049199), cholestasis (PT: 10008635), meningitis (PT: 10027199), sepsis (PT: 10040047), blood bilirubin increased (PT: 10005364), prerenal failure (PT: 10072370), and vein collapse (PT: 10074621).

**TABLE 4:** Signal strength of reports associated with sacituzumab govitecan at the Preferred Terms (PTs) level in the FAERS database.

SOC	Preferred terms (PTs)	PT/N	ROR (95%CI)	PRR (χ2)	IC (IC025)	EBGM (EBGM05)
Blood and lymphatic system disorders	Agranulocytosis	7	4.34 (2.07–9.12)	4.34 (17.97)	2.12 (0.59)	4.33 (2.06)
	Pancytopenia	17	3.53 (2.19–5.68)	3.52 (30.67)	1.81 (0.94)	3.52 (2.18)
	Leukopenia	18	3.91 (2.46–6.21)	3.9 (38.73)	1.96 (1.09)	3.89 (2.45)
	Cytopenia	9	5.22 (2.71–10.04)	5.21 (30.58)	2.38 (0.96)	5.2 (2.7)
	Anaemia	61	3.58 (2.78–4.61)	3.55 (112.12)	1.83 (1.4)	3.55 (2.76)
	Thrombocytopenia	48	4.57 (3.44–6.07)	4.54 (132.66)	2.18 (1.67)	4.54 (3.41)
	Haematotoxicity	10	10.15 (5.45–18.89)	10.13 (81.93)	3.33 (1.59)	10.09 (5.42)
	Neutropenia	204	12.78 (11.11–14.7)	12.41 (2132.56)	3.63 (3.34)	12.34 (10.73)
	Febrile neutropenia	117	16.47 (13.7–19.78)	16.18 (1656.42)	4.01 (3.56)	16.07 (13.38)
	Febrile bone marrow aplasia	18	52.81 (33.06–84.33)	52.66 (890.84)	5.68 (3.14)	51.45 (32.21)
Congenital, familial and genetic disorders	Aplasia	10	41.75 (22.32–78.08)	41.68 (389.66)	5.35 (2.26)	40.92 (21.88)
Eye disorders	Periorbital oedema	4	9.79 (3.66–26.14)	9.78 (31.39)	3.28 (0.53)	9.74 (3.65)
Gastrointestinal disorders	Enterocolitis^a^	4	6.2 (2.32–16.55)	6.2 (17.39)	2.63 (0.31)	6.18 (2.32)
	Diarrhoea	255	3.96 (3.49–4.49)	3.84 (540.28)	1.94 (1.74)	3.84 (3.38)
	Large intestine perforation^a^	5	8.23 (3.42–19.82)	8.23 (31.63)	3.04 (0.72)	8.2 (3.41)
	Enteritis^a^	9	13.65 (7.08–26.29)	13.63 (104.69)	3.76 (1.67)	13.55 (7.03)
	Gastrointestinal toxicity	8	15.98 (7.97–32.04)	15.96 (111.37)	3.99 (1.61)	15.85 (7.9)
	Colitis^a^	48	12.09 (9.1–16.08)	12.01 (482.2)	3.58 (2.87)	11.95 (8.99)
	Neutropenic colitis^a^	39	156.97 (113.33–217.42)	156.02 (5607.6)	7.19 (4.51)	145.71 (105.2)
General disorders and administration site conditions	Asthenia	108	3.17 (2.62–3.83)	3.13 (157.38)	1.65 (1.34)	3.13 (2.59)
	General physical health deterioration	44	3.54 (2.63–4.77)	3.53 (79.68)	1.82 (1.31)	3.52 (2.62)
	Death	303	3.45 (3.07–3.87)	3.33 (501.25)	1.74 (1.55)	3.33 (2.97)
	Performance status decreased	3	9.33 (3–29)	9.33 (22.2)	3.22 (0.15)	9.29 (2.99)
	Hyperthermia	5	7.71 (3.2–18.55)	7.7 (29.05)	2.94 (0.68)	7.68 (3.19)
	Mucosal inflammation	17	7.05 (4.38–11.36)	7.03 (87.74)	2.81 (1.71)	7.01 (4.35)
	Vascular device occlusion^a^	3	51.31 (16.33–161.25)	51.29 (144.54)	5.65 (0.45)	50.14 (15.95)
	Disease progression	622	55.73 (51.26–60.59)	50.44 (29520.03)	5.62 (5.39)	49.32 (45.37)
Hepatobiliary disorders	Hepatic lesion	3	6.63 (2.13–20.59)	6.62 (14.28)	2.72 (0.01)	6.61 (2.13)
	Liver disorder	16	3.89 (2.38–6.36)	3.88 (34.21)	1.96 (1.03)	3.88 (2.37)
	Cholecystitis acute^a^	3	7.61 (2.45–23.65)	7.61 (17.16)	2.92 (0.07)	7.58 (2.44)
	Hepatic cytolysis^a^	9	5.01 (2.6–9.64)	5.01 (28.79)	2.32 (0.92)	5 (2.6)
	Cholestasis^a^	11	6.28 (3.48–11.36)	6.28 (48.65)	2.65 (1.29)	6.26 (3.46)
Infections and infestations	Device related infection^a^	6	4.98 (2.23–11.09)	4.97 (19)	2.31 (0.57)	4.96 (2.23)
	Staphylococcal sepsis^a^	3	8.43 (2.71–26.21)	8.43 (19.56)	3.07 (0.11)	8.4 (2.7)
	Meningitis^a^	4	7.23 (2.71–19.29)	7.22 (21.37)	2.85 (0.39)	7.2 (2.7)
	Sepsis^a^	48	4.77 (3.59–6.34)	4.74 (141.67)	2.24 (1.72)	4.73 (3.56)
	Septic shock^a^	39	9.64 (7.03–13.22)	9.59 (298.97)	3.26 (2.52)	9.55 (6.97)
	Enterocolitis infectious^a^	5	63.29 (26.01–154.05)	63.25 (297.71)	5.94 (1.27)	61.5 (25.27)
	Neutropenic sepsis^a^	26	38.81 (26.31–57.23)	38.65 (937.19)	5.25 (3.44)	38 (25.77)
Investigations	Weight increased	58	2.65 (2.04–3.43)	2.63 (58.85)	1.4 (0.98)	2.63 (2.03)
	Gamma-glutamyltransferase increased^a^	7	4.63 (2.2–9.72)	4.62 (19.83)	2.21 (0.65)	4.61 (2.2)
	Blood bilirubin increased^a^	9	4.65 (2.42–8.94)	4.64 (25.68)	2.21 (0.85)	4.64 (2.41)
	Weight decreased	92	3.17 (2.58–3.9)	3.14 (134.68)	1.65 (1.32)	3.14 (2.55)
	White blood cell count decreased	50	3.96 (3–5.23)	3.94 (109.57)	1.98 (1.49)	3.93 (2.98)
	Haemoglobin abnormal	6	8.47 (3.8–18.88)	8.46 (39.32)	3.08 (0.94)	8.43 (3.78)
	Heart rate increased^a^	48	5.11 (3.84–6.79)	5.08 (157.09)	2.34 (1.81)	5.07 (3.81)
	Neutrophil count decreased	76	16.4 (13.07–20.58)	16.22 (1078.04)	4.01 (3.42)	16.11 (12.84)
	General physical condition abnormal	26	25.4 (17.24–37.41)	25.3 (599.95)	4.65 (3.17)	25.02 (16.99)
	Neutrophil count abnormal	18	56.27 (35.22–89.89)	56.11 (950.01)	5.77 (3.17)	54.73 (34.26)
Metabolism and nutrition disorders	Hypokalaemia	15	3.59 (2.16–5.96)	3.59 (27.95)	1.84 (0.9)	3.58 (2.16)
	Electrolyte imbalance	9	7.6 (3.95–14.63)	7.59 (51.32)	2.92 (1.28)	7.57 (3.93)
	Cell death	9	44.62 (23.05–86.36)	44.56 (375.59)	5.45 (2.13)	43.69 (22.57)
	Weight fluctuation	93	64.3 (52.24–79.13)	63.38 (5550.43)	5.95 (4.92)	61.62 (50.07)
Nervous system disorders	Cholinergic syndrome	5	90.5 (36.98–221.43)	90.43 (424.65)	6.44 (1.3)	86.88 (35.51)
Renal and urinary disorders	Prerenal failure^a^	3	19.66 (6.31–61.3)	19.66 (52.65)	4.28 (0.34)	19.49 (6.25)
Respiratory, thoracic and mediastinal disorders	Pleural effusion	18	3.55 (2.23–5.64)	3.54 (32.82)	1.82 (0.98)	3.54 (2.23)
	Pneumonitis	26	9.17 (6.23–13.49)	9.14 (187.67)	3.19 (2.25)	9.1 (6.19)
Skin and subcutaneous tissue disorders	Skin tightness	3	7.01 (2.26–21.78)	7.01 (15.4)	2.8 (0.04)	6.99 (2.25)
	Alopecia	86	4.37 (3.53–5.41)	4.33 (220.17)	2.11 (1.74)	4.32 (3.49)
Vascular disorders	Lymphoedema	5	6.47 (2.69–15.57)	6.47 (23.04)	2.69 (0.58)	6.45 (2.68)
	Vein collapse^a^	3	26.81 (8.58–83.72)	26.8 (73.6)	4.73 (0.39)	26.48 (8.48)

^a^ Emerging off-label AEs associated with sacituzumab govitecan were identified from the FAERS, database.

### 3.3 Time-to-onset analysis

There were a total of approximately 814 AE reports that reported time of onset. The mean onset time was 49 days, and the median onset time was 14 (IQR, 7–52) days. Our data showed that the most onset time of SG-related AEs was less than 30 days (*n* = 533, 65.48%). Of note, AEs might still have occurred after half a year for SG treatment, with a proportion of 2.56% (Table 2). Furthermore, we statistically analyzed the specific time of occurrence of PTs in each report, as detailed in Table 5, the time to onset of commonly reported AEs associated with SG were neutropenia 10.5 (IQR, 6–17) days, diarrhea 12 (IQR, 7–27) days, anemia 13.5 (IQR, 7–21) days, thrombocytopenia 14 (IQR, 6–39) days, respectively. Notably, the median onset time of heart rate increased was somewhat earlier than the other AEs [7 (IQR, 0–43.5) days], whereas pneumonia occurred relatively later [28 (IQR, 15.25–76.25) days].

**TABLE 5:** Time to onset of AEs associated with sacituzumab govitecan.

AEs	Number^a^	Median (Q1, Q3)
Diarrhoea	130	12 (7, 27)
Neutropenia	94	10.5 (6, 17)
Febrile neutropenia	88	12 (9, 13)
Asthenia	68	14 (5.75, 32.50)
Alopecia	23	6 (0.50, 48.50)
Anaemia	46	13.5 (7, 21)
White blood cell count decreased	34	12 (8, 20.50)
Thrombocytopenia	33	14 (6, 39)
Colitis	41	12 (9, 14)
Neutropenic colitis	29	12 (11,14)
Sepsis	33	12 (10, 21)
Heart rate increased	27	7 (0, 43.50)
Septic shock	30	12 (10, 14.75)
Pneumonitis	14	28 (15.25, 76.25)
Mucosal inflammation	8	11 (7, 51)
Liver disorder	10	10 (4.50, 24.25)
Hypokalaemia	12	7.5 (5.75, 8.75)
Cholestasis	10	10.5 (4.75, 18)

^a^ Indicate the number of reports that record the specific time of AEs occurrence. Q1 = Quartile 1; Q3 = Quartile 3.

## 4 Discussion

Sacituzumab govitecan, a first-in-class ADC targeting Trop-2, showed favorable tolerability and an impressive PFS and OS in patients with heavily pretreated mTNBC (Goldenberg et al., 2015; Bardia et al., 2021a; Bardia et al., 2021b). Since the favorable results of single-agent SG established in the phase 2 TROPHY-U-01 (NCT03547973) trial and phase 3 TROPiCS-02 (NCT03901339) trial, the FDA has granted fast-track approval to SG for patients with locally advanced/metastatic urothelial carcinoma and unresectable locally advanced/metastatic hormone receptor-positive, HER2-negative BC (Tagawa et al., 2021; Rugo et al., 2022b). Subsequently, the clinical practice and prescriptions of SG will inevitably increase. However, safety evidence of SG was limited to clinical trials, which provide only a narrow opinion of severe or even fatal issues (Bardia et al., 2019; Bardia et al., 2021a; Bardia et al., 2021b; Xu et al., 2023). Thus, the purpose of the present study is to decipher potential AEs associated SG to guide the summary of product characteristics, and to delineate the safety spectrum of SG as a reference for clinical medication.

In the present pharmacovigilance study, as shown in Table 2, SG-related AEs were increased significantly from 2020 to 2023, with the 2022 annual report (*n* = 1,257) almost four times as many as in 2021 (*n* = 359). The main reason for the yearly increase in SG-related AE reports may be attributed to the widespread clinical application of SG and the increased awareness of healthcare professionals about post-marketing safety surveillance of drugs. Meanwhile, approximately 88.11% of the AE reports were submitted by health professionals, which might be considered a reliable reporting source. The primary serious outcome of SG is death events, which may be due to the fact that TNBC is the highest recurrence and mortality rates subtype in breast cancer with a 5-year survival 8%–16% (Li et al., 2017; Howard and Olopade, 2021), and therefore mortality events might be more closely related to disease progression.

As described in Tables 3, 4, our disproportionality analyses suggested that the most significant SOCs for SG was congenital, familial and genetic disorders (ROR, 50.02; 95% CI, 28.21–88.68). However, at the SOC level of congenital, familial and genetic disorders, aplasia and uridine diphosphate glucuronosyltransferase 1A1 (UGT1A1) gene mutation is the main reported PTs, which were not recorded in the specification of SG or clinical trials, and more likely to be related to the patient’s development problems or incomplete documentation of AEs information (TRODELVY). Noteworthy, the ROR for PTs of UGT1A1 gene mutation is 4378.02 (396.92–48289.81) high (Supplementary Table S3), this may be one of the major reasons why the SOC signal of congenital, familial and genetic disorders is so significant. The UGT1A1 enzyme plays a crucial role in the detoxification of SN-38 by glucuronidation, and the metabolites it produces are then eliminated from the body primarily by biliary excretion (Ocean et al., 2017; Nelson et al., 2021). The results of previous studies indicated that patients with UGT1A1 homozygous*28/*28 genotype are at a higher risk of developing neutropenia while on SG therapy, and therefore patients known to have UGT1A1*28 homozygosity should be monitored closely (Bardia et al., 2021a; Rugo et al., 2021; Rugo et al., 2022a).

Besides, “investigations” and “blood and lymphatic system disorders” are also common and significant SOC associated with SG. Interestingly, the majority of PTs belonging to “investigations” were hematological AEs (e.g., white blood cell count decreased, haemoglobin abnormal, neutrophil count decreased), which were consistent with the safety results reported in the previous clinical trials (Bardia et al., 2019; Bardia et al., 2021a; Bardia et al., 2021b). In the ASCENT trials, the incidence of febrile neutropenia, leukopenia, anaemia, neutropenia was 6%, 16%, 34%, and 63%, respectively (Bardia et al., 2021b). According to the SG insert, hematotoxicity is the primary AE, which may be caused by DNA double-strand breaks and apoptosis of hematopoietic cell progenitors due to the topoisomerase inhibition payload (SN-38) of SG. However, its overall incidence and severity were much lower than those observed with irinotecan (D’Arienzo et al., 2023; Mathijssen et al., 2001). For neutropenia, granulocyte colony-stimulating factor prophylaxis can be administered to patients at high risk for febrile neutropenia or a moderate risk but with risk factors (Spring et al., 2021). If not managed properly, severe hematologic AEs may lead to complications such as bleeding and possibly secondary infections up to sepsis. Therefore, clinicians should be vigilant in the early assessment and management of SG-related hematologic toxicity.

As for SOC of metabolism and nutrition disorders, hypokalaemia, electrolyte imbalance, and weight fluctuation are the main significant PT signals (Table 4). Previous reports have shown that severe diarrhea leads to loss of body fluids and electrolytes, which can lead to electrolyte imbalance, dehydration and renal insufficiency, and malnutrition (Do et al., 2022). In addition, nausea and vomiting can also lead to systemic complications metabolic imbalances and nutrient depletion (D’Arienzo et al., 2023). Hence, metabolism and nutrition disorders may be secondary complications due to gastrointestinal toxicity induced by SG. Based on safety results from clinical trials (Bardia et al., 2021b; Rugo et al., 2022b), diarrhea occurred in about 60% of SG-treated patients, of whom about 10% had grade 3 events, which is thought to be related to the early dissociation of the drug from its antibody (off-target off-tumor toxicity). As per FDA label and clinical guidelines (Bossi et al., 2018; Bardia et al., 2021b; TRODELVY), if a patient develops acute diarrhea or early cholinergic syndrome (abdominal cramps, diarrhea, sweating, or excessive salivation) during or shortly after the infusion, intravenous atropine 0.4 mg every 15 min for two consecutive doses as needed. If ruled negative, loperamide is recommended in standard therapy.

The most commonly reported AEs during the SG treatment in mTNBC clinical trials were diarrhea, neutropenia, leukopenia, nausea, anemia, constipation, fatigue, alopecia, and vomiting (Bardia et al., 2019; Bardia et al., 2021a; Bardia et al., 2021b; Tagawa et al., 2021). In our disproportionality analyses, the significantly reported PTs for SG were anemia, neutropenia, leukopenia, diarrhea, asthenia, alopecia, which were mostly documented in the manufacturer’s labeling and clinical trials. Furthermore, no significant disproportionate signals were found for nausea, constipation, and vomiting, several frequently reported adverse effects listed in the SG insert. The reason for these discrepancies may be that AEs are fairly common for all drugs in the FAERS database. The large number of reports of AEs associated with multiple drugs may suppress the signal score. Disproportionality requires that drug-specific AEs be reported more frequently (or less frequently). Thus, the absence of a signal does not mean that there are no associated AEs; it simply indicates that these AEs do not appear to be disproportionate (Sakaeda et al., 2013).

Excitingly, as shown in Table 4, our findings also raise some uncommon safety concerns. First, we detected some rare AEs with significant signals, included periorbital oedema, febrile neutropenia, neutropenic colitis, mucosal inflammation, hepatic lesion, gamma-glutamyltransferase increased, cholinergic syndrome, pleural effusion, pneumonitis, and lymphedema, which were reported in the drug’s label and clinical trials (TRODELVY). Second, we found some unexpected PTs with significant signals, including colitis, large intestine perforation, heart rate increased, cholecystitis acute, cholestasis, blood bilirubin increased, meningitis, sepsis, prerenal failure, and vein collapse.

Cardiotoxicity is a known potential AE of HER2-targeting ADC, to date, there were no reports about patients experiencing severe cardiac events related to SG treatment in clinical trials or real-world settings (Bardia et al., 2021a; Bardia et al., 2021b). However, in our disproportionality analyses, the PTs of heart rate increased showed a significant signal (*n* = 48, ROR = 5.11), suggesting that the risk of cardiac AEs related SG shouldn’t be ignored and baseline assessment of risk factors for cardiovascular events remains crucial. On the other hand, anthracycline combined with taxane chemotherapy is the standard adjuvant treatment for TNBC, and cardiotoxicity is the main adverse reaction of anthracyclines (Lee et al., 2021). Thus, the increased heart rate may also be in part a long-term cardiac adverse effect of previous chemotherapy. Anyway, the knowledge of cardiovascular events related SG treatment is urgently needed to be further updated.

In the TROPiCS-02 phase 3 study, neutropenic colitis occurred in 0.5% of patients with hormone receptor-positive/HER2-negative advanced breast cancer, of note, one patient death was related to septic shock from SG associated neutropenic colitis (Rugo et al., 2022d). In the present disproportionality analyses, neutropenic colitis was also detected with a significant signal (*n* = 39, ROR = 156.97). Besides, off-labeling AEs of colitis (*n* = 48, ROR = 12.09) and large intestine perforation (*n* = 5, ROR = 8.23) were detected unexpectedly, which mostly related to the effects of the cytotoxic payload on the mucosal cells (D’Arienzo et al., 2023; Sandmeier et al., 2005). Thus, early recognition and proactive intervention of gastrointestinal toxicity is necessary because these effects can be life-threatening or lead to poor quality of life.

Furthermore, in addition to frequently elevated transaminase, the hepatobiliary toxicity of SG was mainly manifested as acute cholecystitis (ROR, 7.61; 95% CI, 2.45–23.65), cholestasis (ROR, 6.28; 95% CI, 3.48–11.36), blood bilirubin increased (ROR, 4.65; 95% CI, 2.42–8.94) in our present study, which may be related to the excretion of SG through the gallbladder (Ocean et al., 2017). Meanwhile, the meningitis (ROR, 7.23; 95% CI, 2.71–19.29), sepsis (ROR, 4.77; 95% CI, 3.59–6.34), prerenal failure (ROR, 19.66; 95% CI, 6.31–61.3), and vein collapse (ROR, 26.81; 95% CI, 8.58–83.72) were also be detected as potential AEs associated with SG treatment, which may be attributed to the effects of cytotoxic payload to mucosal cells, infection secondary to neutropenia, off-target effects, and infusion reactions, respectively (TRODELVY; D’Arienzo et al., 2023).

The results of the present study indicated that the median time to onset of SG-related AEs was 14 (IQR, 7–52) days, with the majority of AEs occurring within the first month of SG treatment (*n* = 533, 25.76%), and AEs might still have occurred after half a year (Tables 2, 5). Therefore, longer follow-up periods are needed to observe SG-related AEs in future clinical trials. As described in Table 5, the median onset time of boxed warning AEs, neutropenia, and diarrhea, in a real-world setting was 10.5 (IQR, 6–17) days and 12 (IQR, 7–27) days, respectively. This is similar to the median time to onset of neutropenia and diarrhea reported in the safety analyses results of the phase 3 ASCENT trial (neutropenia and diarrhea was 20 days and 12 days, respectively) (Spring et al., 2021). Taken together, these results suggested that clinicians and pharmacists should pay special attention to the labeled and potential AEs of patients, since which can be life-threatening, especially in the first half month of treatment. A better understanding of the real-world safety profile of SG in patients with mTNBC will lead to better compliance, fewer interruptions, and reflection on the desirable PFS and OS.

The main strength of this study is our ability to detect potential AEs that were not observed during the clinical trial stage for SG. As with previous studies based on pharmacovigilance databases, several limitations of the present study need to be addressed. First, due to the voluntary nature of reporting to FAERS database, the incidence and prevalence of AEs cannot be calculated, and underreporting is expected. Second, the presence of reports in the FAERS database is not causally relevant, so the results of the present study are only indicative of potential AEs, which means that clinicians and pharmacists should be vigilant. Third, multiple unmeasured confounders such as potential drug-drug interactions, comorbidities, and drug combinations, which might affect AEs, were not included in the data analysis. Besides, the disproportionality analysis neither quantified risk nor existed causality, but only estimated signal strength, which was only statistically significant. Therefore, prospective clinical trials are still required to verify their causal connection.

## 5 Conclusion

In conclusion, based on real-world data leveraging the FAERS, this study comprehensively investigated and identified AEs highly associated with SG by conducting disproportionality analysis. The AEs detected in the present study were generally consistent with the AEs introduced in the label, and some potential AEs, including colitis, large intestine perforation, heart rate increased, cholecystitis acute, cholestasis, blood bilirubin increased, meningitis, sepsis, prerenal failure, and vein collapse were also be revealed. Moreover, the median onset time of labeled and off-label AEs was reported here to provide vigilance reference for clinicians and pharmacists to optimize medication and manage the safety issues of SG. Given the exploratory character of our work, it is imperative to validate our findings in a prospective study and to elucidate the potential mechanisms and risk factors of AEs for improved risk management.

## Data Availability

The original contributions presented in the study are included in the article/Supplementary Material, further inquiries can be directed to the corresponding author.

## References

[B1] Bardia A. Hurvitz S. A. Tolaney S. M. Loirat D. Punie K. Oliveira M. (2021b). Sacituzumab govitecan in metastatic triple-negative breast cancer. N. Engl. J. Med. 384 (16), 1529–1541. 10.1056/NEJMoa2028485 33882206

[B2] Bardia A. Mayer I. A. Diamond J. R. Moroose R. L. Isakoff S. J. Starodub A. N. (2017). Efficacy and safety of anti-trop-2 antibody drug conjugate sacituzumab govitecan (IMMU-132) in heavily pretreated patients with metastatic triple-negative breast cancer. J. Clin. Oncol. 35 (19), 2141–2148. 10.1200/JCO.2016.70.8297 28291390 PMC5559902

[B3] Bardia A. Mayer I. A. Vahdat L. T. Tolaney S. M. Isakoff S. J. Diamond J. R. (2019). Sacituzumab govitecan-hziy in refractory metastatic triple-negative breast cancer. N. Engl. J. Med. 380 (8), 741–751. 10.1056/NEJMoa1814213 30786188

[B4] Bardia A. Messersmith W. A. Kio E. A. Berlin J. D. Vahdat L. Masters G. A. (2021a). Sacituzumab govitecan, a Trop-2-directed antibody-drug conjugate, for patients with epithelial cancer: final safety and efficacy results from the phase I/II IMMU-132-01 basket trial. Ann. Oncol. 32 (6), 746–756. 10.1016/j.annonc.2021.03.005 33741442

[B5] Bate A. Lindquist M. Edwards I. R. Olsson S. Orre R. Lansner A. (1998). A Bayesian neural network method for adverse drug reaction signal generation. Eur. J. Clin. Pharmacol. 54 (4), 315–321. 10.1007/s002280050466 9696956

[B6] Bossi P. Antonuzzo A. Cherny N. I. Rosengarten O. Pernot S. Trippa F. (2018). Diarrhoea in adult cancer patients: ESMO clinical practice guidelines. Ann. Oncol. 29 (Suppl. l_4), iv126–iv142. 10.1093/annonc/mdy145 29931177

[B7] Carey L. A. Loirat D. Punie K. Bardia A. Diéras V. Dalenc F. (2022). Sacituzumab govitecan as second-line treatment for metastatic triple-negative breast cancer-phase 3 ASCENT study subanalysis. NPJ Breast Cancer 8 (1), 72. 10.1038/s41523-022-00439-5 35680967 PMC9184615

[B8] D’Arienzo A. Verrazzo A. Pagliuca M. Napolitano F. Parola S. Viggiani M. (2023). Toxicity profile of antibody-drug conjugates in breast cancer: practical considerations. EClinicalMedicine 62, 102113. 10.1016/j.eclinm.2023.102113 37554126 PMC10404866

[B9] Do C. Evans G. J. DeAguero J. Escobar G. P. Lin H. C. Wagner B. (2022). Dysnatremia in gastrointestinal disorders. Front. Med. (Lausanne). 9, 892265. 10.3389/fmed.2022.892265 35646996 PMC9136014

[B10] Evans S. J. Waller P. C. Davis S. (2001). Use of proportional reporting ratios (PRRs) for signal generation from spontaneous adverse drug reaction reports. Pharmacoepidemiol Drug Saf. 10 (6), 483–486. 10.1002/pds.677 11828828

[B11] Fang Z. Xu Z. Zhu W. Yu M. Ji C. (2023). A real-world disproportionality analysis of apalutamide: data mining of the FDA adverse event reporting system. Front. Pharmacol. 14, 1101861. 10.3389/fphar.2023.1101861 37342589 PMC10277739

[B12] FDA grants regular approval to sacituzumab, FDA grants regular approval to sacituzumab govitecan for triple-negative breast cancer. News Release, 2021. Available at: https://www.fda.gov/drugs/resources-information-approved-drugs/fda-grants-regular-approval-sacituzumab-govitecan-triple-negative-breast-cancer .

[B13] Garrido-Castro A. C. Lin N. U. Polyak K. (2019). Insights into molecular classifications of triple-negative breast cancer: improving patient selection for treatment. Cancer Discov. 9 (2), 176–198. 10.1158/2159-8290.CD-18-1177 30679171 PMC6387871

[B14] Giaquinto A. N. Sung H. Miller K. D. Kramer J. L. Newman L. A. Minihan A. (2022). Breast cancer statistics, 2022. CA Cancer J. Clin. 72 (6), 524–541. 10.3322/caac.21754 36190501

[B15] Goldenberg D. M. Cardillo T. M. Govindan S. V. Rossi E. A. Sharkey R. M. (2015). Trop-2 is a novel target for solid cancer therapy with sacituzumab govitecan (IMMU-132), an antibody-drug conjugate (ADC). Oncotarget 6 (26), 22496–22512. 10.18632/oncotarget.4318 26101915 PMC4673178

[B16] Gradishar W. J. Moran M. S. Abraham J. Abramson V. Aft R. Agnese D. (2023). NCCN Guidelines® insights: breast cancer, version 4.2023. J. Natl. Compr. Canc Netw. 21 (6), 594–608. 10.6004/jnccn.2023.0031 37308117

[B17] Howard F. M. Olopade O. I. (2021). Epidemiology of triple-negative breast cancer: a review. Cancer J. 27 (1), 8–16. 10.1097/PPO.0000000000000500 33475288 PMC12050094

[B18] Kathpalia M. Sharma A. Kaur N. (2023). Sacituzumab govitecan as a second-line treatment in relapsed/refractory metastatic triple-negative breast cancer patients: a systematic review and meta-analysis. Ann. Pharmacother., 106002802311641. 10.1177/10600280231164110 37026168

[B19] Khosravi-Shahi P. Cabezón-Gutiérrez L. Custodio-Cabello S. (2018). Metastatic triple negative breast cancer: optimizing treatment options, new and emerging targeted therapies. Asia Pac J. Clin. Oncol. 14 (1), 32–39. 10.1111/ajco.12748 28815913

[B20] Kwapisz D. (2022). Sacituzumab govitecan-hziy in breast cancer. Am. J. Clin. Oncol. 45 (7), 279–285. 10.1097/COC.0000000000000919 35728046

[B21] Lee K. J. Wright G. Bryant H. Wiggins L. A. Dal Zotto V. L. Schuler M. (2021). Cytoprotective effect of vitamin D on doxorubicin-induced cardiac toxicity in triple negative breast cancer. Int. J. Mol. Sci. 22 (14), 7439. 10.3390/ijms22147439 34299059 PMC8305038

[B22] Leon-Ferre R. A. Goetz M. P. (2023). Advances in systemic therapies for triple negative breast cancer. BMJ 381, e071674. 10.1136/bmj-2022-071674 37253507

[B23] Li X. Yang J. Peng L. Sahin A. A. Huo L. Ward K. C. (2017). Triple-negative breast cancer has worse overall survival and cause-specific survival than non-triple-negative breast cancer. Breast Cancer Res. Treat. 161 (2), 279–287. 10.1007/s10549-016-4059-6 27888421

[B24] Mathijssen R. H. van Alphen R. J. Verweij J. Loos W. J. Nooter K. Stoter G. (2001). Clinical pharmacokinetics and metabolism of irinotecan (CPT-11). Clin. Cancer Res. 7 (8), 2182–2194. 11489791

[B25] Nelson R. S. Seligson N. D. Bottiglieri S. Carballido E. Cueto A. D. Imanirad I. (2021). UGT1A1 guided cancer therapy: review of the evidence and considerations for clinical implementation. Cancers (Basel) 13 (7), 1566. 10.3390/cancers13071566 33805415 PMC8036652

[B26] Ocean A. J. Starodub A. N. Bardia A. Vahdat L. T. Isakoff S. J. Guarino M. (2017). Sacituzumab govitecan (IMMU-132), an anti-Trop-2-SN-38 antibody-drug conjugate for the treatment of diverse epithelial cancers: safety and pharmacokinetics. Cancer 123 (19), 3843–3854. 10.1002/cncr.30789 28558150

[B27] Park I. H. Im S. A. Jung K. H. Sohn J. H. Park Y. H. Lee K. S. (2019). Randomized open label phase III trial of irinotecan Plus capecitabine versus capecitabine monotherapy in patients with metastatic breast cancer previously treated with anthracycline and taxane: PROCEED trial (KCSG BR 11-01). Cancer Res. Treat. 51 (1), 43–52. 10.4143/crt.2017.562 29458237 PMC6333992

[B28] Rugo H. S. Bardia A. Marmé F. Cortes J. Schmid P. Loirat D. (2022b). Sacituzumab govitecan in hormone receptor-positive/human epidermal growth factor receptor 2-negative metastatic breast cancer. J. Clin. Oncol. 40 (29), 3365–3376. 10.1200/JCO.22.01002 36027558

[B29] Rugo H. S. Bardia A. Marmé F. Cortes J. Schmid P. Loirat D. (2022d). Primary results from TROPiCS-02: a randomized phase 3 study of sacituzumab govitecan (SG) versus treatment of physician’s choice (TPC) in patients (Pts) with hormone receptor–positive/HER2-negative (HR+/HER2-) advanced breast cancer. J. Clin. Oncol. 40 (Suppl. l _17), LBA1001. 10.1200/JCO.2022.40.17_suppl.LBA1001

[B30] Rugo H. S. Tolaney S. M. Loirat D. (2021). Impact of UGT1A1 status on the safety profile of sacituzumab govitecan in the phase 3 ASCENT study in patients with metastatic triple-negative breast cancer [abstract]. Proceedings of the 2020 San Antonio Breast Cancer Virtual Symposium. Cancer Res. 81 (Suppl. 4), PS11–09. 10.1158/1538-7445.SABCS20-PS11-09

[B31] Rugo H. S. Tolaney S. M. Loirat D. Punie K. Bardia A. Hurvitz S. A. (2022a). Safety analyses from the phase 3 ASCENT trial of sacituzumab govitecan in metastatic triple-negative breast cancer. NPJ Breast Cancer 8 (1), 98. 10.1038/s41523-022-00467-1 36038616 PMC9424318

[B32] Sakaeda T. Tamon A. Kadoyama K. Okuno Y. (2013). Data mining of the public version of the FDA adverse event reporting system. Int. J. Med. Sci. 10 (7), 796–803. 10.7150/ijms.6048 23794943 PMC3689877

[B33] Sandmeier D. Chaubert P. Bouzourene H. (2005). Irinotecan-induced colitis. Int. J. Surg. Pathol. 13 (2), 215–218. 10.1177/106689690501300215 15864388

[B34] Shu Y. Chen J. Ding Y. Zhang Q. (2023a). Adverse events with risankizumab in the real world: postmarketing pharmacovigilance assessment of the FDA adverse event reporting system. Front. Immunol. 14, 1169735. 10.3389/fimmu.2023.1169735 37256136 PMC10225532

[B35] Shu Y. Ding Y. He X. Liu Y. Wu P. Zhang Q. (2023b). Hematological toxicities in PARP inhibitors: a real-world study using FDA adverse event reporting system (FAERS) database. Cancer Med. 12 (3), 3365–3375. 10.1002/cam4.5062 35871395 PMC9939145

[B36] Spring L. M. Nakajima E. Hutchinson J. Viscosi E. Blouin G. Weekes C. (2021). Sacituzumab govitecan for metastatic triple-negative breast cancer: clinical overview and management of potential toxicities. Oncologist 26 (10), 827–834. 10.1002/onco.13878 34176192 PMC8488774

[B37] Starodub A. N. Ocean A. J. Shah M. A. Guarino M. J. Picozzi V. J. Vahdat L. T. (2015). First-in-Human trial of a novel anti-trop-2 antibody-SN-38 conjugate, sacituzumab govitecan, for the treatment of diverse metastatic solid tumors. Clin. Cancer Res. 21 (17), 3870–3878. 10.1158/1078-0432.CCR-14-3321 25944802 PMC4558321

[B38] Szarfman A. Machado S. G. O'Neill R. T. (2002). Use of screening algorithms and computer systems to efficiently signal higher-than-expected combinations of drugs and events in the US FDA's spontaneous reports database. Drug Saf. 25 (6), 381–392. 10.2165/00002018-200225060-00001 12071774

[B39] Tagawa S. T. Balar A. V. Petrylak D. P. Kalebasty A. R. Loriot Y. Fléchon A. (2021). TROPHY-U-01: a phase II open-label study of sacituzumab govitecan in patients with metastatic urothelial carcinoma progressing after platinum-based chemotherapy and checkpoint inhibitors. J. Clin. Oncol. 39 (22), 2474–2485. 10.1200/JCO.20.03489 33929895 PMC8315301

[B40] TRODELVY Prescribing information. 2023. Available at: https://www.gilead.com/-/media/files/pdfs/medicines/oncology/trodelvy/trodelvy_pi.pdf (Accessed February, 2023).

[B41] Twelves C. Jove M. Gombos A. Awada A. (2016). Cytotoxic chemotherapy: still the mainstay of clinical practice for all subtypes metastatic breast cancer. Crit. Rev. Oncol. Hematol. 100, 74–87. 10.1016/j.critrevonc.2016.01.021 26857987

[B42] van Puijenbroek E. P. Bate A. Leufkens H. G. Lindquist M. Orre R. Egberts A. C. (2002). A comparison of measures of disproportionality for signal detection in spontaneous reporting systems for adverse drug reactions. Pharmacoepidemiol Drug Saf. 11 (1), 3–10. 10.1002/pds.668 11998548

[B43] Winer E. P. Lipatov O. Im S. A. Goncalves A. Muñoz-Couselo E. Lee K. S. (2021). Pembrolizumab versus investigator-choice chemotherapy for metastatic triple-negative breast cancer (KEYNOTE-119): a randomised, open-label, phase 3 trial. Lancet Oncol. 22 (4), 499–511. 10.1016/S1470-2045(20)30754-3 33676601

[B44] Xu B. Ma F. Wang T. Wang S. Tong Z. Li W. (2023). A Phase IIb, single arm, multicenter trial of sacituzumab govitecan in Chinese patients with metastatic triple-negative breast cancer who received at least two prior treatments. Int. J. Cancer 152 (10), 2134–2144. 10.1002/ijc.34424 36621000

