# Supplementary material for: Post-marketing safety surveillance of sacituzumab govitecan: an observational, pharmacovigilance study leveraging FAERS database
Source: Front Pharmacol. 2023 Nov 10;14:1283247. doi: 10.3389/fphar.2023.1283247 (PMC10667432; doi:10.3389/fphar.2023.1283247)
Supplement: Supplementary file 1 [file DataSheet1.docx]

**Supplementary Table 1** Two-by-two contingency table for disproportionality analysis.

|  | **Number of target adverse events** | **Number of other adverse events** | **Total** |
| --- | --- | --- | --- |
| **Target drugs** | a | b | a + b |
| **Other drugs** | c | d | c + d |
| **Total** | a + c | b + d | a + b + c + d |

**Supplementary Table** **2** Signal strengths of PTs at neoplasms benign, malignant and unspecified (incl cysts and polyps), injury, poisoning and procedural complications, product issues and surgical and medical procedures of the sacituzumab govitecan.

| **SOC** | **Preferred Terms (PTs)** | **PT/N** | **ROR (95%CI)** | **PRR (χ2)** | **IC (IC025)** | **EBGM (EBGM05)** |
| --- | --- | --- | --- | --- | --- | --- |
| Neoplasms benign, malignant and unspecified (incl cysts and polyps) | Breast cancer recurrent | 3 | 10.54 (3.39-32.79) | 10.54 (25.77) | 3.39 (0.19) | 10.49 (3.37) |
|  | Metastases to liver | 16 | 8.73 (5.34-14.28) | 8.71 (108.86) | 3.12 (1.88) | 8.68 (5.31) |
|  | Metastasis | 8 | 11.43 (5.7-22.91) | 11.42 (75.67) | 3.51 (1.44) | 11.37 (5.67) |
|  | Breast cancer metastatic | 11 | 10.58 (5.85-19.14) | 10.56 (94.79) | 3.39 (1.72) | 10.52 (5.81) |
|  | Metastases to meninges | 4 | 16.81 (6.28-44.97) | 16.8 (58.99) | 4.06 (0.71) | 16.68 (6.24) |
|  | Metastases to skin | 3 | 27.48 (8.8-85.84) | 27.47 (75.57) | 4.76 (0.39) | 27.14 (8.69) |
|  | Metastases to central nervous system | 31 | 24.51 (17.19-34.95) | 24.39 (687.94) | 4.59 (3.29) | 24.14 (16.93) |
|  | Triple negative breast cancer | 15 | 228.48 (134.17-389.08) | 227.95 (3069.62) | 7.69 (3.14) | 206.54 (121.29) |
| Injury, poisoning and procedural complications | Product preparation error | 10 | 4.05 (2.18-7.54) | 4.05 (22.92) | 2.02 (0.79) | 4.04 (2.17) |
|  | Intentional dose omission | 15 | 4.34 (2.61-7.2) | 4.33 (38.35) | 2.11 (1.12) | 4.32 (2.6) |
|  | Product preparation issue | 10 | 9.56 (5.13-17.8) | 9.55 (76.2) | 3.25 (1.55) | 9.51 (5.11) |
|  | Inappropriate schedule of product administration | 194 | 6.32 (5.48-7.29) | 6.16 (840.29) | 2.62 (2.37) | 6.15 (5.33) |
|  | Counterfeit product administered | 3 | 22.19 (7.11-69.21) | 22.18 (60.06) | 4.46 (0.36) | 21.97 (7.04) |
|  | Dose calculation error | 3 | 33.68 (10.77-105.37) | 33.67 (93.65) | 5.05 (0.42) | 33.17 (10.6) |
| Product issues | Product temperature excursion issue | 5 | 6.52 (2.71-15.7) | 6.52 (23.3) | 2.7 (0.58) | 6.5 (2.7) |
| Surgical and medical procedures | Therapy change | 8 | 8.8 (4.39-17.63) | 8.79 (55) | 3.13 (1.27) | 8.76 (4.37) |
|  | Radiotherapy | 3 | 20.4 (6.54-63.59) | 20.39 (54.8) | 4.34 (0.35) | 20.21 (6.48) |
|  | Palliative care | 6 | 31.07 (13.87-69.57) | 31.04 (172) | 4.94 (1.45) | 30.62 (13.67) |

| **SOC** | **Preferred Terms (PTs)** | **N** | **ROR (95% CI)** | **PRR (χ2)** | **IC(IC025)** | **EBGM(EBGM05)** |
| --- | --- | --- | --- | --- | --- | --- |
| Congenital, familial and genetic disorders | Aplasia | 10 | 41.75(22.32-78.08) | 41.68(389.66) | 5.35(2.26) | 40.92(21.88) |
|  | UGT1A1 gene mutation | 2 | 4378.02 (396.92-48289.81) | 4376.66(2916.44) | 10.51(-0.62) | 1459.55(132.32) |

**Supplementary Table** **3** Signal strengths of PTs at SOC of congenital, familial and genetic disorders of the sacituzumab govitecan.
